# Neuroprotective effect of nerolidol against neuroinflammation and oxidative stress induced by rotenone

**DOI:** 10.1186/s12868-016-0293-4

**Published:** 2016-08-22

**Authors:** Hayate Javed, Sheikh Azimullah, Salema B. Abul Khair, Shreesh Ojha, M. Emdadul Haque

**Affiliations:** 1 Department of Biochemistry, College of Medicine and Health Sciences, United Arab Emirates University, PO Box 17666, Al Ain, UAE; 2 Department of Pharmacology and Therapeutics, College of Medicine and Health Sciences, United Arab Emirates University, PO Box 17666, Al Ain, UAE

**Keywords:** Parkinson disease, Rotenone, Neurodegeneration, Oxidative stress, Nerolidol

## Abstract

**Background:**

Parkinson disease (PD) is a movement disorder affecting 1 % of people over the age of 60. The etiology of the disease is unknown; however, accumulating evidence suggests that mitochondrial defects, oxidative stress, and neuroinflammation play important roles in developing the disease. Current medications for PD can only improve its symptoms, but are unable to halt its progressive nature. Although many therapeutic approaches are available, new drugs are urgently needed for the treatment of PD. Thus, the present study was undertaken to investigate the neuroprotective potential of nerolidol, a sesquiterpene alcohol, on a rotenone-induced experimental model of PD, where male Wistar rats intraperitoneally received rotenone (ROT) at a dose of 2.5 mg/kg of body weight once daily for 4 weeks.

**Results:**

Nerolidol, which has antioxidant and anti-inflammatory properties, was injected intraperitoneally at 50 mg/kg of body weight, once daily for 4 weeks, and at 30 min prior to ROT administration. ROT administration significantly reduced the activities of antioxidant enzymes superoxide dismutase (SOD) and catalase (CAT), and the level of the antioxidant tripeptide glutathione (GSH). Moreover, ROT increased the levels of the lipid peroxidation product malondialdehyde (MDA), proinflammatory cytokines (IL-1β, IL-6, and TNF-α), and inflammatory mediators (COX-2 and iNOS) in rat brain tissues. Immunostaining of brain tissue sections revealed a significant increase in the number of activated astrocytes (GFAP) and microglia (Iba-1), along with the concomitant loss of dopamine (DA) neurons in the substantia nigra pars compacta and dopaminergic nerve fibers in the striatum of ROT-treated rats. As expected, nerolidol supplementation to ROT-injected rats significantly increased the level of SOD, CAT, and GSH, and decreased the level of MDA. Nerolidol also inhibited the release of proinflammatory cytokines and inflammatory mediators. Finally, nerolidol treatment prevented ROT-induced glial cell activation and the loss of dopaminergic neurons and nerve fibers, and ultimately attenuated ROT-induced dopaminergic neurodegeneration.

**Conclusion:**

Our findings are the first to show that the neuroprotective effect of nerolidol is mediated through its anti-oxidant and anti-inflammatory activities, which strongly supports its therapeutic potential for the treatment of PD.

## Background

Parkinson disease is a neurodegenerative disorder that is characterized by a progressive loss of dopamine (DA) neurons in the substantia nigra pars compacta (SNc) and dopaminergic nerve terminal fibers of the striatum that are enriched in the DA transporter [1]. As a result of dopaminergic neurodegeneration, the level of the DA neurotransmitter (which mediates the control of movement) is significantly reduced in the striatum. Therefore, dopaminergic neurodegeneration leads to impairments in movement, a resting tremor, rigidity, and a disturbance in gait. Although the etiology for dopaminergic neuronal degeneration in PD is not completely understood, current evidence suggests that oxidative stress and neuroinflammation play key roles in the pathogenesis of PD [2, 3].

Rotenone (ROT), an inhibitor of mitochondrial complex I, has been widely used as a herbicide/pesticide in agriculture. A ROT challenge, due to its complex I inhibitory property, induces pathological features in animals that are similar to those seen in human PD patients. Thus, ROT-treated animals represent a promising animal model with construct validity [4–6]. The ROT model recapitulates most of the pathological features observed in human PD pathogenesis, including the loss of DA neurons in SNc, and enhanced oxidative stress and neuroinflammation in the nigrostriatal dopaminergic pathway [2, 6–8]. Moreover, a ROT challenge to rodents induced the formation of α-synuclein cytoplasmic inclusions in DA neurons, Lewy pathology, DJ-1 acidification and translocation, proteasomal dysfunction, and nigral iron accumulation [9]. Therefore, the ROT-treated rat is a suitable model for investigating novel therapeutic agents targeting oxidative stress and neuroinflammation in PD.

Currently available drugs alleviate the symptoms of PD, but are inadequate in terms of halting the progression of the disease. Additionally, motor complications during advanced stages of the disease, the adverse effects of the available drugs, and non-motor symptoms remain huge challenges for long-term therapy. Therefore, newer therapeutic agents and approaches are urgently needed to stop or delay the progressive nature of the disease [10]. PD is intimately connected to excessive oxidative stress that damages brain areas and accelerates the process of neurodegeneration, especially in the nigrostriatal area. This process is an important breakpoint for controlling the disease. Consequently, in addition to other therapeutic approaches, treatment with antioxidants is gradually gaining importance in the pharmacotherapy of PD.

**Fig. 1:**
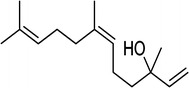
Chemical structure of nerolidol

Plants are a potential source of novel drugs to benefit mankind. Much research effort has focused on discovering new antioxidant compounds from plants. An increasing number of studies have shown that essential oils obtained from medicinal plants exhibit a variety of biological properties, such as anticonvulsant [11], analgesic [12], anxiolytic [13], antidepressant [14], antioxidant [15], and anti-inflammatory [16] properties. Some of these effects are frequently attributed to terpenes, which are chemical components that are present in these essential oils [11, 17]. Nerolidol (NRD) [3,7,11-trimethyl-1,6,10-dodecatrien-3-ol] is a sesquiterpene alcohol (Fig. 1) that is found in essential oils from plants such as *Baccharis dracunculifolia* [18], *Amaranthus retroflexus* [19], and *Canarium schweinfurthii* [20]. Nerolidol is an aliphatic sesquiterpene that is commonly used in cosmetics, perfumes, shampoos, and soaps [21], as well as non-cosmetic products (cleansers and detergents). Essential oils from *Ferula fukanensis* containing nerolidol have been shown to prevent nitric oxide (NO) production and NO-induced gene expression, suggesting its plausible use as an antioxidant agent. NRD is hydrophobic in nature with an XlogP3 value of 4.6 (>2.5 is needed for efficient transport across blood brain barrier), and thus NRD can cross easily the blood brain barrier. NRD significantly reduces the level of lipid peroxidation and nitrite content in the hippocampus of mice, which protects against oxidative stress [15], suggesting an important antioxidant property for NRD. A recent study reported that NRD reduces interleukin-1β (IL-1β) production in lipopolysaccharide-induced peritoneal macrophages [16]. Additionally, NRD shows potent anti-inflammatory activity through the suppression of IL-1β and tumor necrosis factor-alpha (TNF-α) levels in an experimental mouse model of pain [16]. Therefore, the main objective of the present study was to investigate the anti-oxidative and anti-inflammatory effects of NRD on ROT-induced neurodegeneration in rats.

## Methods

### Drugs and chemicals

Polyclonal rabbit anti-cyclooxygenase-2 (COX-2), anti-inducible nitric oxide synthase (iNOS), and anti-glial fibrillary acidic protein (GFAP) antibodies were purchased from Abcam, Cambridge, MA, USA. Anti-ionized calcium-binding adaptor molecule-1 (Iba-1) polyclonal rabbit antibody was procured from Wako Chemicals, Richmond, VA, USA. Polyclonal rabbit anti-tyrosine hydroxylase antibody was obtained from Novus Biologicals, Littleton, CO, USA. Alexa fluor 488 conjugated secondary goat anti-rabbit antibodies were purchased from Life Technologies, Grand Island, NY, USA. Biotinylated secondary anti-rabbit antibody was purchased from Jackson Immunoresearch, West Grove, PA, USA. Nerolidol was purchased from Santa Cruz Biotechnology Inc., CA, USA. ROT and the assay kit for reduced glutathione (GSH) and other reagents of analytical grade were purchased from Sigma-Aldrich, St. Louis, MO, USA.

### Experimental animals

We used male Wistar rats at 6–7 months old (weighing 280–300 g) from the Animal Research Facility of the College of Medicine and Health Sciences, United Arab Emirates University, UAE. Prior to the start of the experiment, a maximum of 4 rats were housed per cage, and rats were acclimatized for 1 week to the laboratory conditions. The cages of the animals were changed twice a week. The animals were housed and kept under standard laboratory light and dark cycle conditions. The animals were fed with commercially available rodent food and water ad libitum. All experiments were conducted between 0900 and 1500 hours. The Animal Ethics Committee of the United Arab Emirates University, UAE, approved the experimental protocol for animal experimentation.

### Experimental design

To induce the PD in rats, ROT (2.5 mg/kg body weight) was administered intraperitoneally (i.p.) once daily for 4 weeks. The regimen used in the current study for the induction of Parkinsonism in rats was adopted as reported earlier [22]. Briefly, ROT was first dissolved in dimethyl sulfoxide (DMSO) to prepare a 50X stock solution and stored at −80 °C for further use. Before injection, the stock solution of ROT was thawed and further diluted in sunflower oil to obtain a final concentration of 2.5 mg/mL. To evaluate its neuroprotective efficacy, NRD was initially diluted in olive oil to obtain a final concentration of 50 mg/2 mL, and injected i.p. 30 min prior to ROT administration at a dose of 50 mg/kg of body weight once daily for 4 weeks. The dose of NRD was selected based on dose–response studies (data not shown) as well as earlier reports [15, 23]. Rats that were used as controls received an equivalent volume of vehicle only. The animals were sacrificed 48 h after the last injection of NRD or ROT or both in combination to completely eliminate these drugs from the body. The rats were divided into 4 experimental groups where each group consisted of 8 animals; the groups were named as follows:

- Group I, the vehicle-injected control group (CONT).
- Group II, the rotenone-injected and vehicle-treated group (ROT).
- Group III, the rotenone-injected and NRD-treated group (ROT + NRD).
- Group IV, the NRD-only injected group (NRD).

### Tissue collection

At the end of the experiment, the animals from all groups were anaesthetized with pentobarbital (40 mg/kg of body weight) and a cardiac perfusion was performed using 0.01 M of phosphate-buffered saline (PBS) pH 7.4 to completely clear the blood. The skull of the rat was open to quickly isolate the brain. The brain was placed on an ice-plate and cut along the midline to separate the 2 cerebral hemispheres. For biochemical assay, the midbrain and striatum regions of the brain were isolated from 1 hemisphere and immediately fresh frozen in liquid nitrogen for further use. The other hemisphere of the brain was incubated in 4 % paraformaldehyde solution for 48 h and subsequently exchanged with 10 % sucrose solution containing 0.1 M of phosphate buffer (PB) 3 times daily for 3 consecutive days at 4 °C prior to cryostat sectioning.

### Sample preparation for biochemical assay

For biochemical assay, the midbrain tissue of each animal was homogenized in KCl buffer (10-mM Tris–HCl, 140-mM-NaCl, 300-mM KCl, 1-mM EDTA, and 0.5 % Triton X-100) at pH 8.0 supplemented with protease and phosphatase inhibitor, keeping the samples on ice. The tissue homogenates of each sample were centrifuged at 14,000×*g* for 20 min at 4 °C to obtain the post-mitochondrial supernatant (PMS) fraction. This PMS fraction was used to estimate the levels of the antioxidant enzyme GSH, lipid peroxidation product, and proinflammatory cytokines using spectrophotometric measurements and an enzyme-linked immunosorbent assay (ELISA) following a standard protocol, as reported earlier [22].

### Assay for lipid peroxidation

A malondialdehyde (MDA) assay kit procured from Northwest Life Science (Vancouver, WA, USA) was used to determine the amount of lipid peroxidation, as reported earlier [22]. Briefly, the samples or standards (250 µL) were incubated in the presence of acid reagent (250 µL) and thiobarbituric acid (250 µL) and vortexed vigorously. Samples were further incubated for 60 min at 60 °C followed by centrifugation at 10,000×*g* for 2–3 min. The reaction mixture was then aseptically transferred without disturbing the pellet to a cuvette and the absorbance was recorded at 532 nm. The concentration of MDA was calculated using a standard curve and expressed as µM MDA/mg protein.

### Estimation of GSH

A GSH kit was used to estimate the GSH level, as reported earlier [22]. Briefly, the samples were first deproteinized with 5 % 5-sulfosalicylic acid solution and centrifuged to remove the precipitated protein. The supernatant was used to measure the GSH level. Samples and standards of different concentrations (10 µL) were added in each well of a 96-well plate and incubated for 5 min with the working mixture (150 µL; assay buffer + 5,5′-dithiobis (2-nitrobenzoic acid) + glutathione reductase). Diluted NADPH solution (50 µL) was added to each well and mixed properly. The absorbance of the samples was measured at 412 nm with kinetics capability for 5 min using a microplate reader. The results were expressed as µM GSH/mg protein.

### Assay for antioxidant enzymes activity

The activity of antioxidant enzymes such as superoxide dismutase (SOD) and catalase (CAT) were determined following the manufacturer’s instructions of a kit (Cayman Chemicals Company, Ann Arbor, MI, USA), as reported earlier [22]. The CAT activity was expressed as nmol/min/mg protein and the SOD activity was expressed as U/mg protein.

### Assay for pro-inflammatory cytokines

Commercially available ELISA kits for IL-1β, IL-6, and TNF-α were purchased from R&D systems, Minneapolis, MN, USA. The level of IL-1β, IL-6, and TNF-α were estimated as described earlier [22]. The results were expressed as pg/mg protein.

### Immunohistochemistry for tyrosine hydroxylase (TH) expression

The one hemisphere of the brains collected from each animal were serially cut after fixation and processed for immunohistochemical analysis. Briefly, 14 μm-thick coronal brain sections were cut at the level of the striatum and SNc using a cryostat (Leica, Wetzlar, Germany) for the immunohistochemical analysis of TH. Sections were washed twice with 0.01 M of PBS, pH 7.4, and then incubated with blocking reagent (10 % normal goat serum in PBS containing 0.3 % Triton-X 100) for 1 h. Next, the sections were incubated overnight with a primary polyclonal rabbit antibody against TH (1:500) at 4 °C. Sections were washed and incubated with biotinylated secondary anti-rabbit (1:1000) antibody for 1 h at room temperature. The brain sections were incubated with avidin–biotin complex (Vector Laboratories Ltd. Burlingame, CA, USA) and 3,3′ diaminobenzidine (DAB) to visualize and analyze the TH immunoreactivity. Finally, the sections were coverslipped using DPX mounting medium. The slides were then viewed under a light microscope (Olympus, Hamburg, Germany) and images were acquired for analysis.

### Immunofluorescence staining of GFAP and Iba-1

Immunofluorescence staining was performed using 14 µm-thick striatum sections to quantify the number of activated GFAP-positive astrocytes and Iba-1-positive microglia. The brain sections were first washed twice with PBS and incubated with blocking reagent (10 % normal goat serum in PBS containing 0.3 % Triton-X 100) for 1 h. The sections were then incubated overnight with the primary polyclonal rabbit antibodies ant-GFAP (1:1000) and anti-Iba-1 (1:1000) at 4 °C. The sections were washed and incubated with fluorescent secondary anti-rabbit Alexa fluor 488 antibody for 1 h at room temperature. Sections were then washed, mounted on slides, and coverslipped using the mounting medium Fluoroshield (Sigma Aldrich, St. Louis, MO, USA). The images were captured using a fluorescence microscope EVOS FL (Thermo Fisher Scientific, Waltham, MA, USA).

### Assessment of TH-immunopositive (TH+) dopamine (DA) neurons in the SNc and TH-immunoreactive (TH-ir) DA nerve fibers in the striatum

To evaluate the ROT-induced neurodegeneration and neuroprotective effect of NRD, the total number of TH+ DA neurons at 3 different anatomical levels of the SNc (−4.8, −5.04, and −5.28 mm of the bregma) were counted. The average number of TH+ neurons were calculated and converted as a percentage with reference to the control. The loss of striatal fibers was evaluated by measuring the intensity of TH-ir dopaminergic fibers in the striatum using Image J software (NIH, Bethesda, MD, USA). The intensity of the TH-immunoreactive nerve fibers in 3 different fields of brain sections (3 sections per animal) within the striatal region (adjacent to 0.3 mm of the bregma) was measured to examine the DA nerve fibers loss. An average of the 3 sections was calculated and presented as a percentage with reference to the values of the control group. The intensity of the overlying cortex area was used as the background measurement and subtracted from the value generated from the striatum. An investigator that was ‘blind’ to the experimental groups counted the TH+ DA neurons and determined the immunoreaction intensity of the TH fibers.

### Assessment of activated astrocytes and microglia in the striatum

Three coronal sections from a comparable anatomical level of striatum from each animal were used to analyze and count the number of activated astrocytes and microglia. Activated astrocytes and microglia were considered for counting based on the intense immunoreactivity of GFAP- and Iba-1-labeled cells, whose characteristic morphological features include hypertrophied and extended glial processes. The total number of activated astrocytes and microglia were counted from 3 randomly chosen fields of an equal area in each section using the Image J software (NIH, Bethesda, MD, USA), and the results were presented as a percentage.

### Western blot analysis of COX-2 and iNOS expression

Western blot analysis was carried out to measure the level of COX-2 and iNOS expression in different groups of animals following the protocol, as reported earlier [22]. Briefly, striatal tissues isolated from each animal were homogenized in radioimmuno-precipitation buffer with protease and phosphatase inhibitors. The cell lysates were then centrifuged at 15,000 rpm for 20 min. The supernatant containing cytoplasmic fractions was isolated, and protein concentration was measured as mentioned below. The cytoplasmic fraction containing equal amounts of protein (35 μg) were loaded and separated using 10 % SDS–polyacrylamide gel electrophoresis. The proteins were then transferred onto a PVDF membrane and incubated overnight at 4 °C with specific primary rabbit polyclonal antibodies against COX-2 (1:1000) and iNOS (1:500). The membrane was washed and then incubated with horseradish peroxidase-conjugated secondary anti-rabbit antibody. The protein recognized by the antibody was visualized using an enhanced chemiluminescence Pico kit (Thermo Fisher Scientific, Rockford, IL, USA). The blots were stripped and re-probed for β-actin (1:5000; monoclonal mouse, Millipore, MA, USA) as a loading control. The intensity of the bands was measured using densitometry and quantified using Image J software (NIH, Bethesda, USA).

### Protein estimation

The concentration of protein in each sample was estimated using the Pierce BCA protein assay kit (Thermo Fisher Scientific, Rockford, IL, USA) following the manufacturer’s instructions.

### Statistical analyses

The data were expressed as the mean value ± SEM. The data were analyzed with Graph Pad (InStat software, La Jolla, CA, USA) using a one-way analysis of variance (ANOVA) followed by a Tukey’s test to determine the statistical significance between various groups. For all of the tests, the criterion for any statistically significant difference was set at *p* < 0.05.

## Results

### NRD administration prevents the loss of DA neurons in the SNc and DA nerve fibers in the striatum

**Fig. 2:**
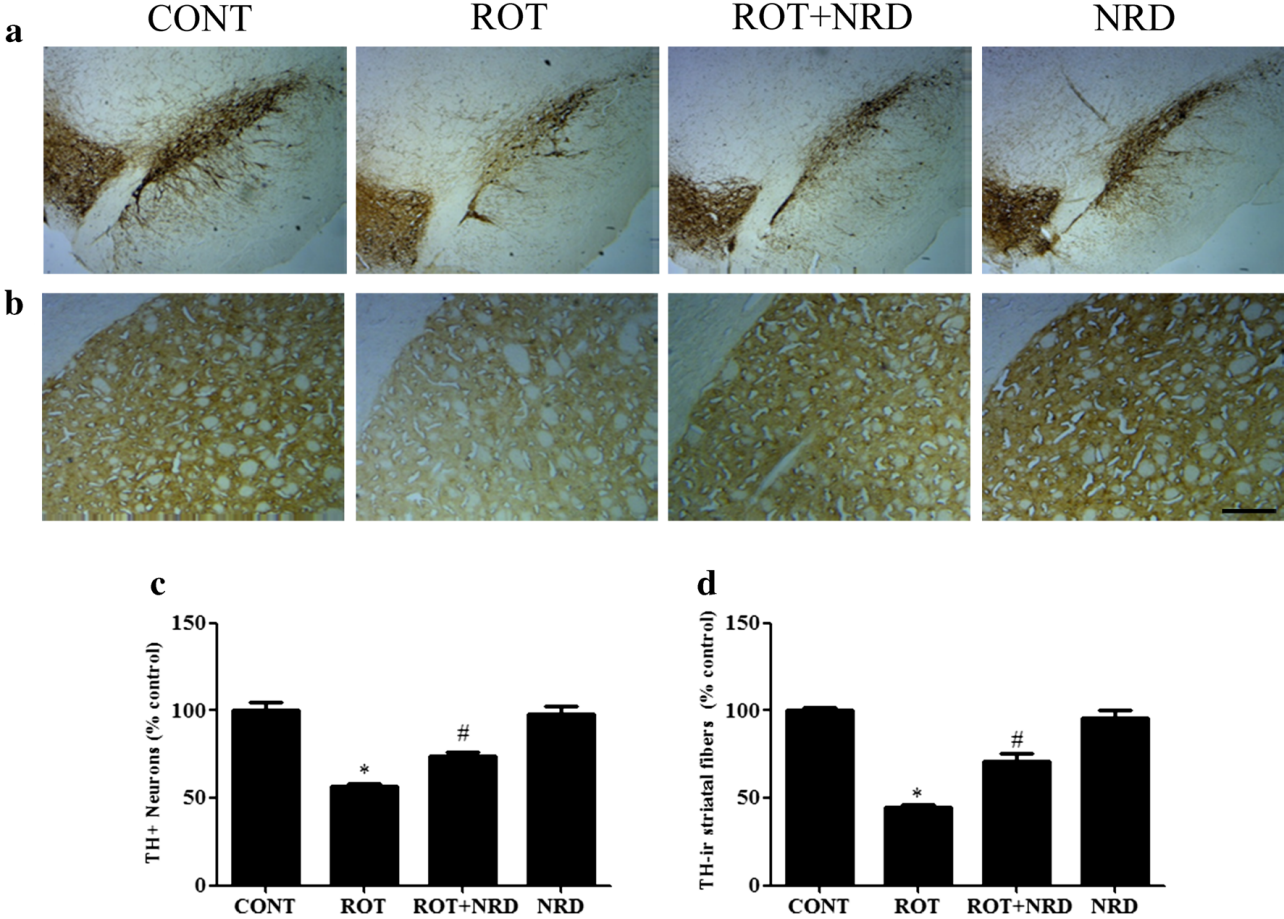
Expression of tyrosine hydroxylase (TH)-immunopositive (TH+) dopamine (DA) neurons in the substantia nigra pars compacta (SNc) and TH-immunoreactive (TH-ir) dopaminergic nerve fibers in the striatum. The *scale bar* is 100 µm. **a** The number of TH+ neurons was decreased in the SNc region of the rotenone (ROT)-injected rats compared to the control (CONT) group. In contrast, nerolidol (NRD) administration rescued the TH+ neurons in the ROT + NRD injected rats compared to the ROT rats. **b** The expression of TH-ir fibers in the striatum of the CONT, ROT, ROT + NRD, and NRD-only group of rats. **c** The number of TH+ positive DA neurons in the SNc was counted from each group. A significant decrease (*p < 0.05) in the number of DA neurons was observed in the SNc of the ROT group compared to the CONT group. NRD treatment significantly (^#^p < 0.05) protected the DA neurons from the ROT-induced neuronal death. No significant difference was observed in the DA neurons of the CONT and NRD-only group. Values are expressed as percent mean ± SEM (n = 3). **d** A significant decrease (*p < 0.05) in the TH-ir fibers was observed in the ROT group compared to the CONT group. NRD treatment significantly inhibited (^#^p < 0.05) the loss of TH-ir fibers in the ROT + NRD group compared to the ROT group. The CONT rats and NRD-only-injected rats did not show a remarkable loss of TH-ir fibers

To investigate the beneficial effect of NRD on DA neurodegeneration in ROT-injected rats, TH immunohistochemical analysis was performed to assess the expression of healthy TH+ DA neurons in the SNc and the TH-ir DA nerve fibers density in the striatum. The results show that chronic ROT treatment caused a significant loss (p < 0.05) of DA neurons in the SNc area as expected when compared to vehicle-injected control rats (Fig. 2a, c). In contrast, NRD supplementation prior to ROT injection in rats provided significant (p < 0.05) protection to DA neurons when compared to only ROT injection of rats. The DA neurons of the SNc project their nerve terminals to the striatum where they are highly enriched with the dopamine transporter (DAT). Degeneration of DA neurons in the SNc decreases DAT expression in the striatum, which further confirms the neuronal loss. Therefore, we examined whether the loss of DA neurons in the SNc area is correlated with DA-terminal loss by measuring the intensity of TH-ir dopaminergic nerve terminal fibers of the striatum. A significant decrease (p < 0.05) was observed in the intensity of TH-ir fibers in ROT-injected animals when compared to vehicle-treated controls. However, NRD supplementation prior to ROT administration significantly increased (p < 0.05) TH-ir intensity in nerve terminal fibers, suggesting that NRD has a potent neuroprotective effect against ROT-induced dopaminergic neurodegeneration (Fig. 2b, d).

### NRD treatment suppresses excessive lipid peroxidation and ameliorates GSH levels

**Fig. 3:**
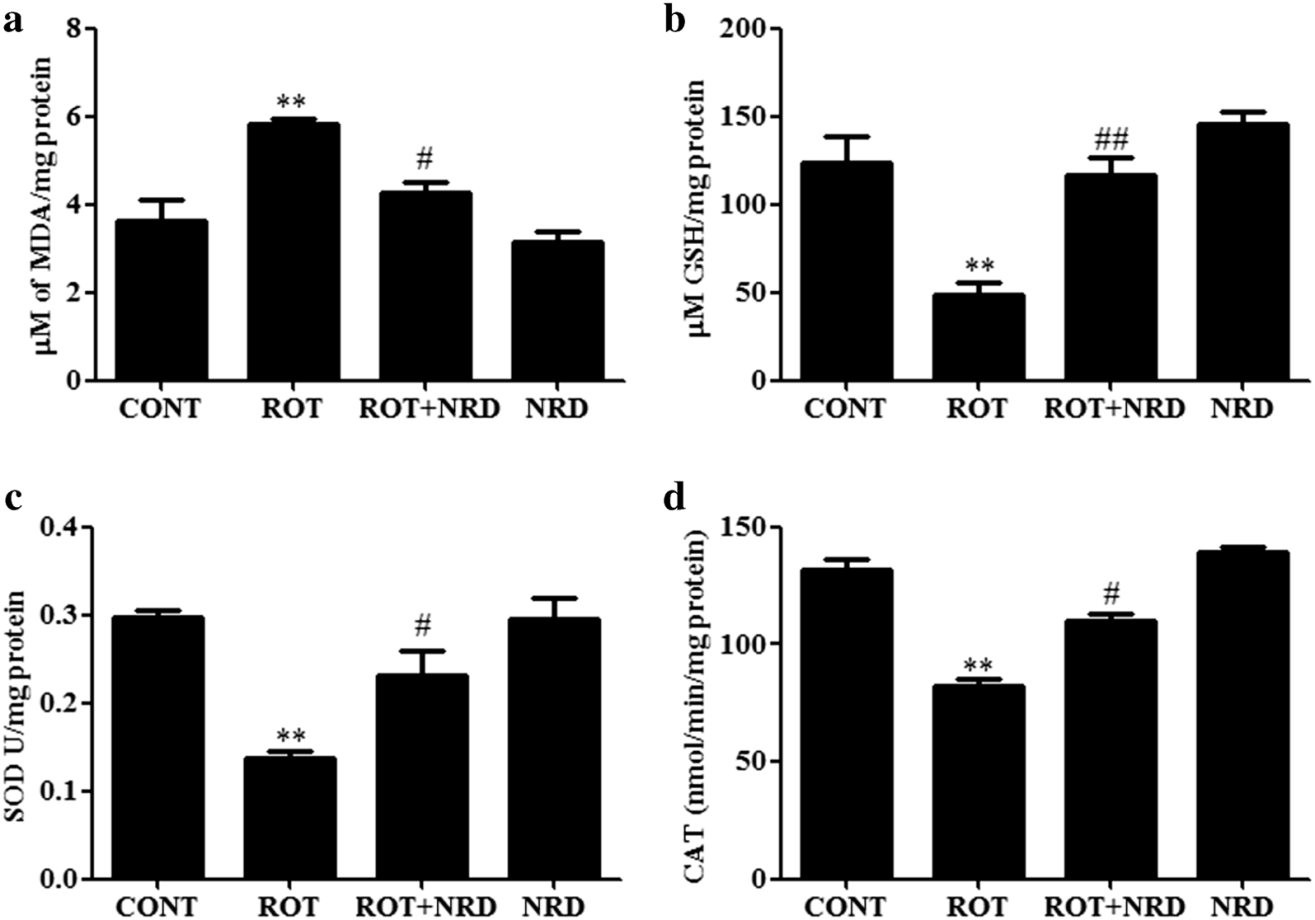
Quantification of malondialdehyde (MDA), glutathione (GSH), superoxide dismutase (SOD) and catalase (CAT) in midbrain tissue. Rotenone (ROT) exposure induced a significant increase (**p < 0.01) in the MDA level (**a**) and a decrease in the GSH level (**b**) in the midbrain of the ROT-challenged rats compared to the vehicle injected control (CONT) rats. NRD supplementation to the ROT-administered rats significantly decreased (^#^p < 0.05) the level of MDA, and increased (^##^p < 0.01) the level of GSH. ROT injection also significantly decreased (**p < 0.01) the activity of SOD (**c**) and CAT (**d**) compared to the CONT rats. NRD supplementation significantly inhibited (^#^p < 0.05) the ROT-induced decrease in SOD and CAT activity compared to the ROT-injected rats. Values are expressed as the mean ± SEM (n = 6–8)

Next, we tested whether NRD mediated the neuroprotective effect that we observed in our model is due to its antioxidant activity because NRD is an antioxidant. To clarify this issue, the role of NRD in ROT-induced neurodegeneration was investigated by examining the level of MDA, a marker for lipid peroxidation in the midbrain region. As shown in Fig. 3a, ROT treatment resulted in a massive amount of MDA production in the ROT group of animals when compared to control rats. Treatment with NRD significantly abolished (p < 0.05) the elevated MDA production. Increased MDA production due to oxidative stress correlated with a decrease in the bioavailability of the tripeptide antioxidant GSH in midbrain cells. The ROT-challenged rats also showed a significantly decreased (p < 0.05) level of GSH compared to vehicle-injected control rats (Fig. 3b). Supplementation with NRD prior to ROT administration significantly attenuated (p < 0.05) the level of GSH compared to the ROT-injected rats. Taken together, these results suggest that the neuroprotective effect of NRD on ROT-induced neurodegeneration might be mediated through its anti-oxidative effect.

### NRD reverses the ROT-induced decrease in the activity of antioxidant enzymes

We also measured the activity of antioxidant enzymes SOD and CAT. ROT administration significantly decreased (p < 0.01) SOD and CAT activity when compared with the vehicle-injected control group. However, NRD supplementation significantly increased (p < 0.05) the activities of SOD (Fig. 3c) and CAT (Fig. 3d) when compared with the ROT-injected animals. Control animals did not show any significant changes in the activities of SOD and CAT when compared to the NRD-only-injected rats.

### Neuroprotective effects of NRD involves the inhibition of glial cell activation

**Fig. 4:**
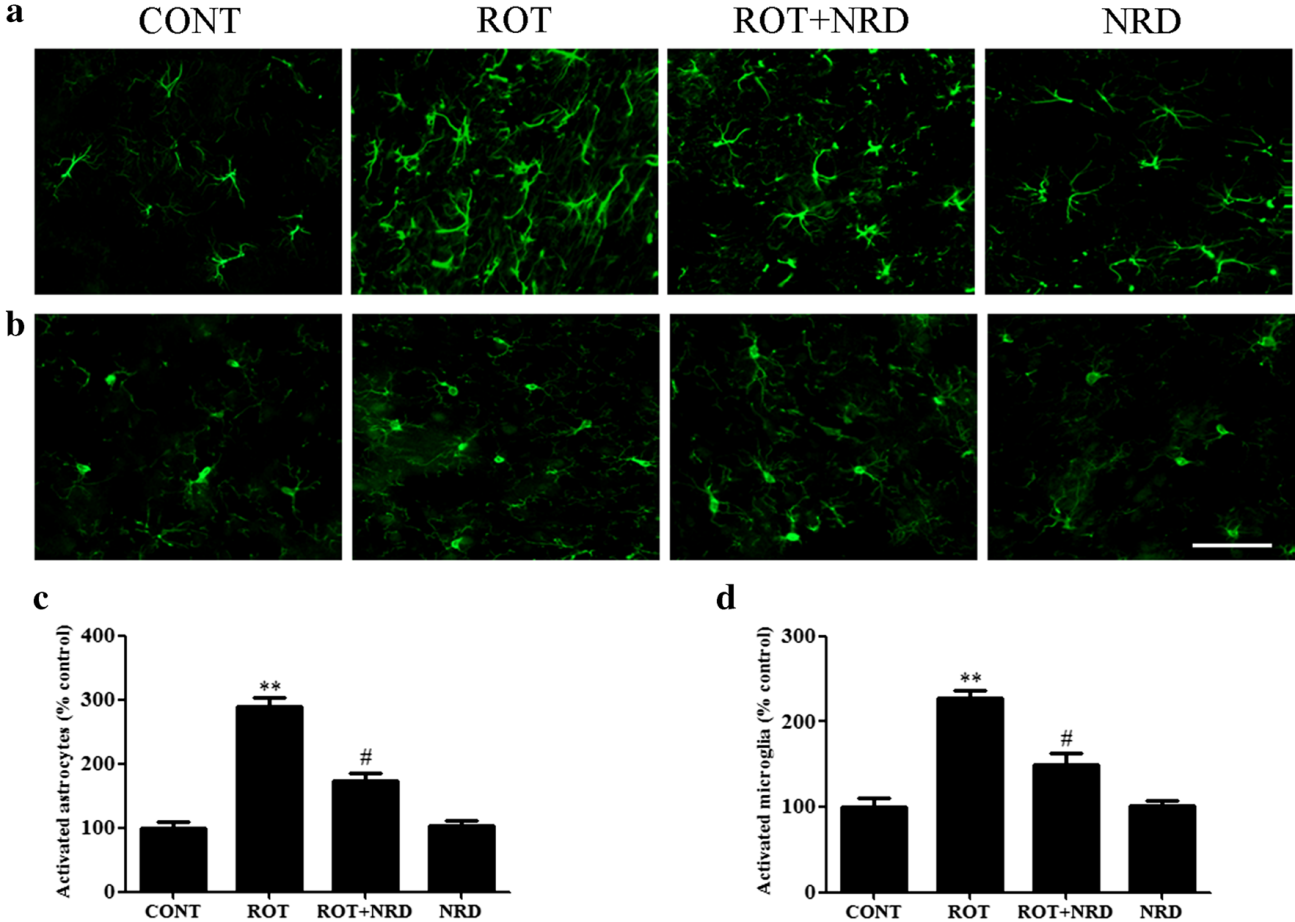
Striatal glial fibrillary acidic protein (GFAP)-positive astrocytes and ionized calcium binding adaptor molecule-1 (Iba-1)-positive microglia. Profound expression of GFAP-positive astrocytes (*green*, **a**) and Iba-1-positive microglia (*green*, **b**) was found in the ROT-administered rats compared to the vehicle-injected CONT rats. In contrast, NRD supplementation to the ROT injected rats showed moderate staining of GFAP and Iba-1 compared to the ROT-injected rats (*scale bar* = 200 µm). Quantitative analysis of activated astrocytes (**c**) and microglia (**d**) revealed a significant increase (**p < 0.01) in the number of activated astrocytes and microglia in the ROT group rats compared to the CONT group. However, NRD supplementation significantly reduced (^#^p < 0.05) the number of activated astrocytes and microglia in the ROT + NRD group compared to the ROT group. CONT rats and NRD-only-injected rats did not show any marked difference in the activation of astrocytes and microglia. Values are expressed as the percent mean ± SEM (n = 3)

Glial cell (astrocyte and microglia) activation following ROT administration has been observed, and is considered to be an index of the inflammatory response. We observed an increase in the expression of GFAP and Iba-1, which are markers of activated astrocytes and microglia, respectively, upon ROT administration. Immunofluorescence staining showed a significant increase (p < 0.01) in the number of activated astrocytes and microglia in ROT-injected animals compared to the vehicle-injected control animals (Fig. 4a–d). Interestingly, NRD treatment to the ROT-injected rats significantly decreased (p < 0.05) the number of activated astrocytes and microglia compared to the ROT-injected rats. Animals injected with NRD alone did not show any remarkable activation of astrocytes and microglia when compared to control animals.

### NRD attenuates the ROT-induced release of proinflammatory cytokines

**Fig. 5:**
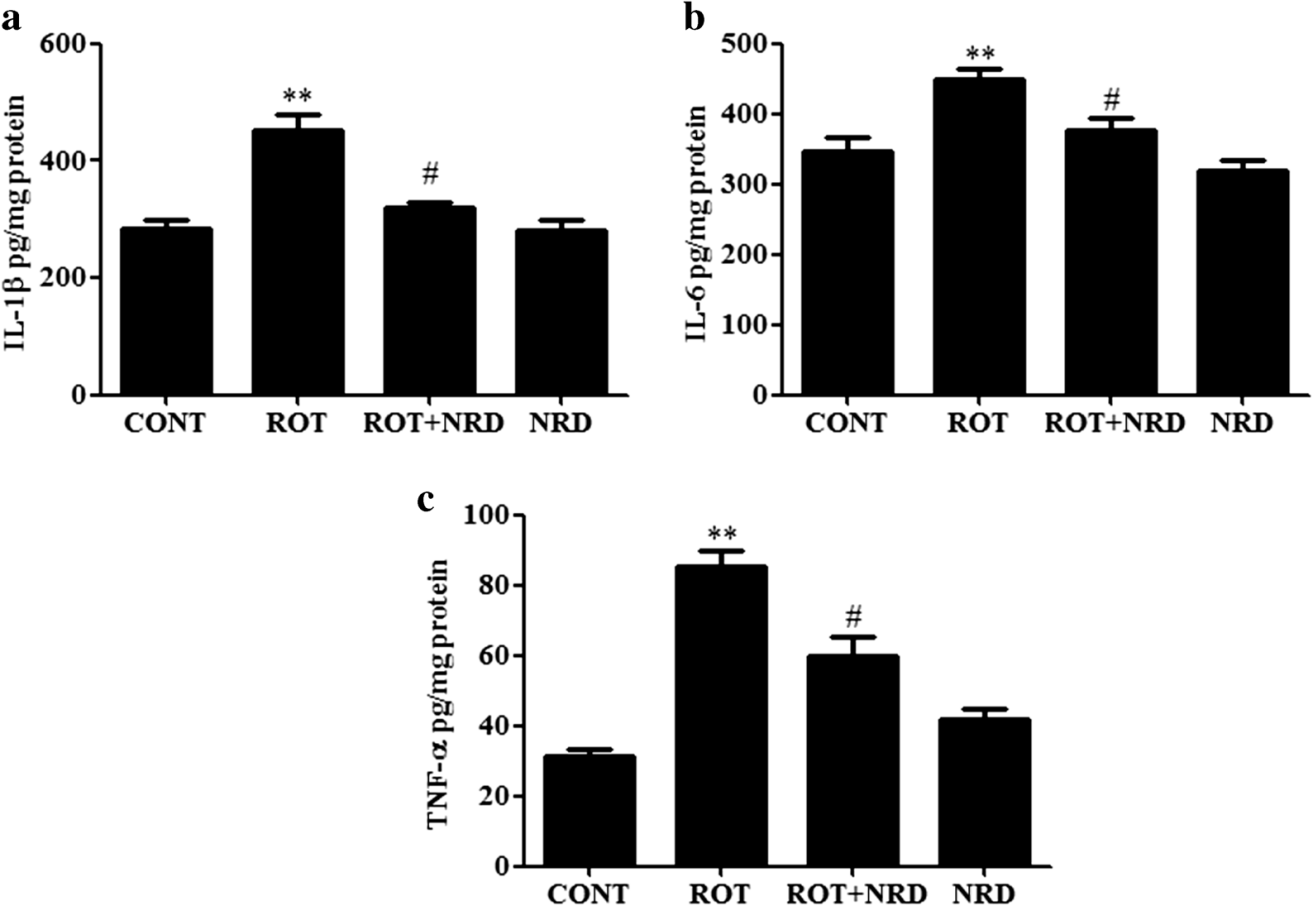
Proinflammatory cytokines in the midbrain tissue of the CONT, ROT, ROT + NRD, and NRD-only groups. The level of IL-1β (**a**), IL-6 (**b**), and TNF-α (**c**) was significantly increased (**p < 0.01) in the ROT-treated group when compared to the CONT group. However, NRD treatment significantly decreased (^#^p < 0.05) the ROT-induced increase of these proinflammatory cytokines in the ROT + NRD group. No significant differences were observed in these cytokines between the CONT and NRD-only group. Values are expressed as the mean ± SEM (n = 6–7)

The persistent activation of astrocytes and microglia accompanied by the sustained secretion of inflammatory mediators is thought to be involved in the neuronal injury in PD. Therefore, the release of proinflammatory cytokines such as IL-1β, IL-6, and TNF-α in response to ROT exposure was examined and quantified using ELISA. A significant increase (p < 0.01) in the IL-1β, IL-6, and TNF-α level was observed in the ROT-injected animals when compared to the vehicle-injected control animals (Fig. 5a–c). However, NRD supplementation to the ROT-administered animals significantly decreased (p < 0.05) the level of these proinflammatory cytokines when compared to the ROT-injected animals (Fig. 5a–c). Animals injected with NRD alone did not show any significant changes in the level of analyzed proinflammatory cytokines when compared to control animals.

### Effect of NRD on the expression of COX-2 and iNOS

**Fig. 6:**
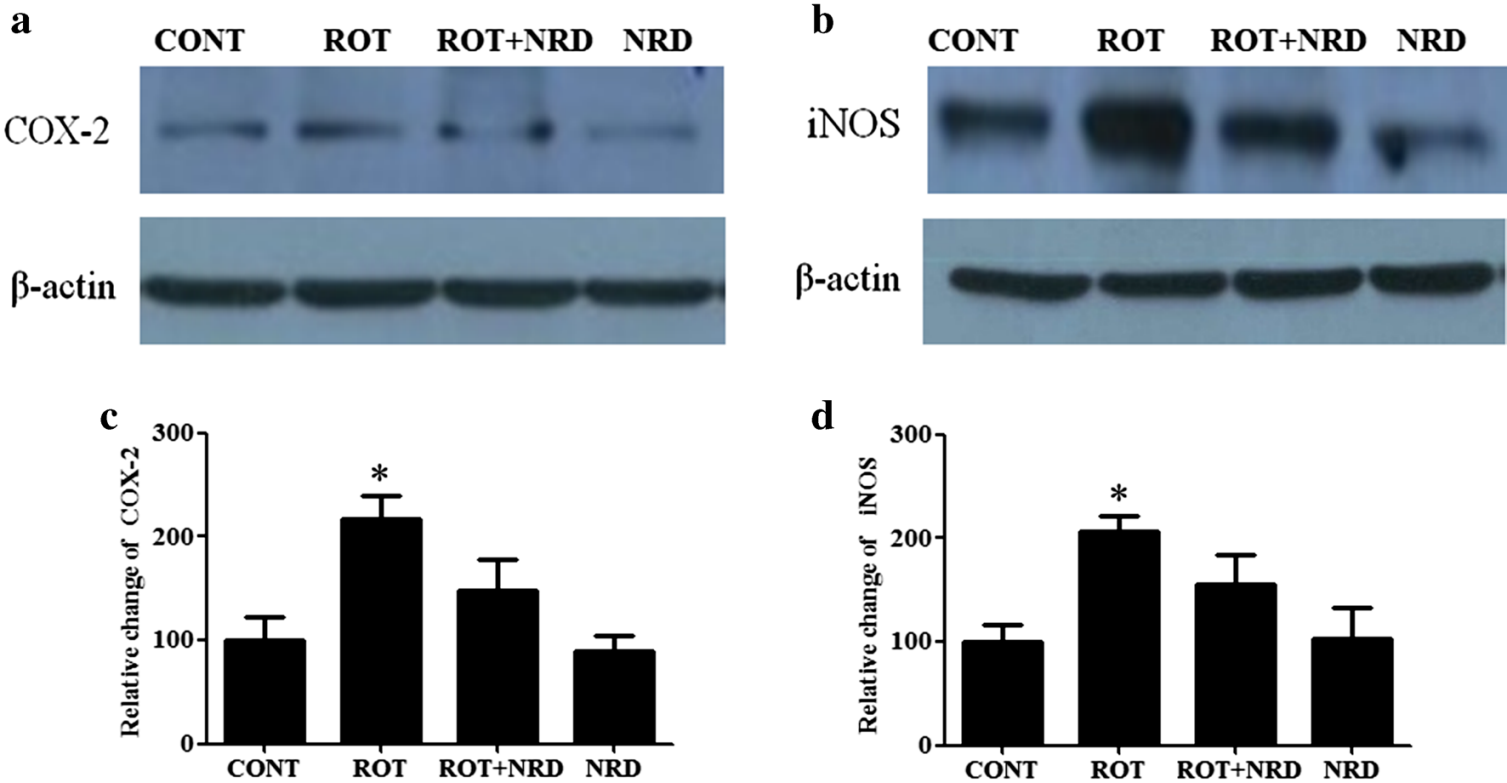
Western blot analysis of COX-2 and iNOS expression in striatal tissue. COX-2 (**a**) and iNOS (**b**) expression levels were determined using Western blotting in the striatum. The ROT-administered group showed significant increase (*p < 0.05, 217.42 vs. 100 %) in the COX-2 level compared to the CONT group. NRD supplementation to the ROT-injected rats remarkably decreased the expression level of COX-2 (148.16 vs. 217.42 %) compared to the ROT group (**c**). Likewise, iNOS expression was also significantly increased (*p < 0.05, 206.35 vs. 100 %) in the ROT group compared to the CONT group. NRD treatment markedly decreased the iNOS expression (156.16 vs. 206.35 %) compared to the ROT group (**d**). NRD-only treatment decreased (89.9 vs. 100 %) COX-2 expression, while an increase (102.76 vs. 100 %) in iNOS expression was observed when compared to the CONT group (n = 3)

The expressions of COX-2 and iNOS were also examined using Western blot in tissue lysates that were isolated from the striatum region (Fig. 6a, b). A significant increase (p < 0.05) in COX-2 expression (Fig. 6c) was observed in ROT-treated animals (217.42 vs. 100 %) when compared to the vehicle-injected control group of animals. However, following treatment with NRD in the ROT-administered rats, a remarkable reduction in the level of COX-2 (148.16 vs. 217.42 %) was observed when compared to the ROT-treated rats. ROT administered animals also showed a significant increase (p < 0.05, 206.35 vs. 100 %) in iNOS expression (Fig. 6d) when compared to the control animals. Interestingly, NRD supplementation to the ROT-injected rats modestly decreased (156.16 vs. 206.35 %) iNOS induction when compared to the ROT-treated rats. Treatment with only NRD decreased COX-2 expression by 10.1 % (89.9 vs. 100 %), whereas a slight increase of 2.76 % (102.76 vs. 100 %) in iNOS expression was observed when compared to the control group.

## Discussion

PD is one of the most common progressive neurodegenerative disorders with a complex pathogenesis. The current hypothesis suggests that mitochondrial dysfunction, oxidative stress, and neuroinflammation play a crucial role in the development of PD. Systemic administration of ROT to rats has been shown to induce behavioral and pathological symptoms of PD, such as motor dysfunction, dopaminergic neurodegeneration, oxidative stress, inflammation, and α-synucleinopathy [6, 22, 24, 25]. In the present study, the ROT-induced PD model was used to investigate the neuroprotective potential of the NRD phytochemical, which recently showed potential antioxidant activity [15]. ROT at a dose of 2.5 mg/kg of body weight once daily was used for 4 weeks to induce dopaminergic neurodegeneration. Our results show the novel potential benefits of NRD in attenuating dopaminergic neurodegeneration, improving antioxidant enzymes, and inhibiting inflammatory mediators and lipid peroxidation in the brain.

Tyrosine hydroxylase (TH) is a rate-limiting enzyme that catalyzes the synthesis of the DA neurotransmitter, which is synthesized in the dopaminergic neurons of the SNc area. DA is stored in synaptic vesicles and, in response to stimuli, released in the striatum to exert its physiological function [26]. In PD patients, more than 70 % of the dopaminergic neurons are dead resulting in the retraction of dopaminergic nerve terminals in the striatum and depletion of DA in the striatum [27]. Therefore, the death of dopaminergic neurons in the SNc is believed to decrease the level of striatal dopamine. The loss of dopaminergic neurons in the SNc and reduced density of striatal nerve terminals are considered to be the pathological hallmark of human PD [28].

In the present study, immunohistochemical examination of TH revealed that ROT causes a significant loss of TH+ dopaminergic neurons in the SNc area and the density of dopaminergic nerve terminal fibers in the striatum, which concurs with previous reports [22, 25]. Interestingly, NRD supplementation to ROT-treated rats significantly protected against the ROT-induced loss of dopaminergic neurons in the SNc and reduced striatal nerve fiber density in the striatum. This result indicates that NRD has neuroprotective effects that prevent dopaminergic neuron loss and striatal nerve terminal retraction in ROT-induced PD in rats. A convincing number of studies have shown that dopaminergic neurons exist in a state of constant oxidative stress, as brain cells have a low antioxidant defense capacity and a tendency to generate reactive oxygen species due to the presence of highly oxidizable DA [29, 30]. In our study, NRD administration protected against ROT-induced dopaminergic neurodegeneration possibly through its strong antioxidant action, as NRD shows efficient antioxidant activity in the hippocampus [15].

Defects in complex-I of the mitochondrial electron transport chain leads to a massive release of free radicals, and consequently, cellular death [31]. ROT, which is an inhibitor of complex-I of the mitochondrial electron transport chain, upon systemic administration to rats, causes the loss of ATP production and subsequently generates reactive oxygen species [30]. Furthermore, reactive oxygen species induce the oxidation of polyunsaturated fatty acid in a process called lipid peroxidation, which is characterized by the formation of MDA, a major product of lipid peroxidation that forms adducts with DNA bases and proteins, thereby causing cellular damage. The increased level of oxidative damage to DNA, proteins, and lipids, and decreased level of antioxidants have been reported in the brain of PD patients [4]. Significantly increased levels of MDA in the ROT-treated rats were observed compared to the vehicle-injected control rats. Interestingly, NRD supplementation to the ROT-injected rats significantly lowered the levels of MDA compared to the ROT-injected rats. Notably, NRD ameliorates against oxidative stress due to lipid peroxidation in the mouse hippocampus [15]. The inhibition of lipid peroxidation by NRD in the current study may have been mediated through the detoxification of peroxy radicals and reactive oxygen species, which further supports an antioxidative role for NRD.

GSH is an important antioxidant and has a crucial role in scavenging hydrogen peroxide. The decreased levels of GSH in the brain might indicate a state of oxidative stress. Decreased levels of GSH have been observed in surviving dopaminergic neurons of the SNc of PD patients compared to age-matched controls [32]. In the present study, the GSH level was significantly lower in ROT-treated rats than in control rats. Decreased levels of GSH lead to oxidative damage to DNA, protein, and lipids in PD [32–34]. Administration of NRD significantly increased GSH levels compared to the ROT injected and vehicle-treated rats (Group II). The effect of NRD on GSH may involve the direct antioxidant effects of NRD or the prevention of ROT-induced GSH oxidation.

SOD and CAT are the main antioxidant enzymes involved in detoxifying free radicals. The loss of SOD activity further contributes to an increase in oxidative stress in ROT-treated rats. Complex-I of the mitochondrial respiratory chain is the major source of superoxide radicals through inhibition of the electron transport chain [28]. The diminished activity of SOD would be detrimental in the scenario when superoxide radical production is increased. Our present data showed decreased activity of SOD in ROT-challenged rats compared to control rats. The administration of NRD was beneficial, because this treatment efficiently restored the SOD activity in ROT-injected rats. This result corroborated a previous report showing an increase in SOD activity after NRD administration [15]. CAT is an enzyme that is responsible for catalyzing the decomposition of H_2_O_2_. The maintenance of a normal metabolism of reactive oxygen species is important for proper cell function in different body parts [35, 36]. In the present study, CAT activity was significantly diminished in ROT-injected rats, while the administration of NRD significantly reversed the decreased activity of CAT in NRD-supplemented rats. NRD supplementation reduced oxidative damage, as observed by the decrease in lipid peroxidation, restoration of the GSH level, and activities of the antioxidant enzymes (SOD and CAT) following ROT administration in rats.

Furthermore, ROT-treated animals showed an increase in neuroinflammation that is triggered and sustained through various mechanisms. Dysfunction of complex-I of the mitochondrial respiratory chain due to ROT treatment increased the release of reactive oxygen species, which can trigger the activation of glial cells [37]. A large number of studies report the involvement of neuroinflammation in PD, which is largely characterized by an accumulation of activated microglia [38]. The activation of astrocytes and microglia results in increased expression of GFAP and Iba-1, respectively. Microglial activation also causes NO· overproduction via iNOS induction [39]. Robust microgliosis and increased expression of iNOS have also been reported in postmortem brain samples of PD patients [40, 41]. Proinflammatory cytokines such as IL-1β, IL-6, and TNF-α, and enzymes such as iNOS and COX-2 appear to be involved in DA toxicity, and have been observed in the cerebrospinal fluid and postmortem brain tissue samples of PD patients [40, 42–45].

In addition, inflammatory processes that are associated with the increased expression of COX-2 and iNOS are involved in the cascade of deleterious events that leads to neurodegeneration in PD [46]. In line with this report, here we showed an increase in activated astrocytes and microglia with a concomitantly enhanced level of the proinflammatory cytokines IL-1β, IL-6, and TNF-α in ROT-treated rats, which was significantly attenuated following NRD supplementation. Additionally, NRD supplementation normalized the expression of COX-2 and iNOS in the ROT-injected rats. Therefore, the present findings further suggest that NRD exerts its anti-inflammatory effects through inhibiting the increased level of COX-2 and iNOS, as well as proinflammatory cytokines. The NRD-mediated suppression of inflammation, as observed in the present study, could be facilitated by the strong anti-inflammatory activity of NRD that has been reported in other studies [47, 48]. NRD has also been shown to inhibit the lipopolysaccharide-induced release of pro-inflammatory cytokines in bone marrow-derived dendritic cells, and diminish the induction of inflammatory mediators in an in vivo acute model of inflammation [49, 50]. Oxidative stress and neuroinflammation play major roles in different neurodegenerative diseases such as stroke, Alzheimer’s disease, and PD [51]. Drugs with anti-oxidative and anti-inflammatory characteristics are considered effective therapeutic agents for these neurodegenerative diseases [52]. Therefore, NRD, with its effective antioxidant and anti-inflammatory properties, may be responsible for the neuroprotection in the ROT-induced rat model of PD.

## Conclusions

In conclusion, our study suggests that NRD supplementation ameliorates the ROT-induced dopaminergic neurodegeneration through its antioxidant and anti-inflammatory activities. The present results highlight the therapeutic potential of NRD in neurodegenerative diseases including PD. However, further study is required to fully understand the molecular mechanisms of how NRD potentiates antioxidant and anti-inflammatory activities.

## Abbreviations

CAT: catalase
DA: dopamine
DAT: dopamine transporter
GSH: glutathione
MDA: malondialdehyde
NRD: nerolidol
PD: Parkinson disease
ROT: rotenone
SNc: substantia nigra pars compacta
SOD: superoxide dismutase
TH+: tyrosine hydroxylase immunopositive
TH-ir: tyrosine hydroxylase immunoreactive
